# Supplementary material for: WordCommentsAnalyzer: A windows software tool for qualitative research
Source: F1000Res. 2018 Sep 4;7:536. Originally published 2018 May 3. [Version 2] doi: 10.12688/f1000research.14819.2 (PMC6137411; doi:10.12688/f1000research.14819.2)
Supplement: Supplementary file 3 [file f1000research-7-17614-s0002.tgz › b2a79496-4ada-47c4-9682-a99ce867836d.zip!/Eight Iranian Health Journals/Journal of Research in Rehabilitation Sciences _10.docx]

© Journal of Research in Rehabilitation Sciences . This work is licensed under CC BY-NC 4.0 (https://creativecommons.org/licenses/by-nc/4.0/).

Volume & Issue: دوره 12، شماره 1: 1395

Title: تأثير تغييرات وابسته به سن و آگاهی صريح و ضمنی بر عملكرد و تحكيم توالی حركتی تركيبی

http://jrrs.mui.ac.ir/index.php/jrrs/article/view/2585

مقدمه: هدف از انجام مطالعه حاضر، بررسی تأثیر تغییرات وابسته به سن و آگاهی صریح و ضمنی بر عملکرد و تحکیم توالی حرکتی ترکیبی بود. مواد و روش‌ها: در این پژوهش، 96 پسر سالم از نظر سیستم عصبی و راست دست با دامنه سنی 18-6 سال، به صورت در دسترس انتخاب شدند. از تکلیف SRT (Serial reaction time) برای بررسی مقایسه‌ای عملکرد صریح و ضمنی آزمودنی‌ها در دو مؤلفه زمان و دقت پاسخ استفاده گردید. کل مداخله شامل دو فاز (اکتساب و تحکیم) بود که طی آن‌ها عملکرد گروه‌ها با یکدیگر مقایسه شد. مرحله اکتساب شامل انجام 8 بلوک و مرحله تحکیم شامل انجام 2 بلوک بود. داده‌ها با استفاده از آزمون Mixed ANOVA در یک طرح، (بلوک) 8 × (گروه سنی) 4 × (نوع یادگیری) 2 مورد تجزیه و تحلیل قرار گرفت. همچنین، جهت انجام مقایسات زوجی از آزمون Bonferroni استفاده گردید. یافته‌ها: در متغیر زمان پاسخ، بلوک (031/0 = P) و سن (001/0 = P) تأثیر معنی‌داری را نشان داد، اما شرایط یادگیری معنی‌دار نبود (431/0 = P). در متغیر دقت پاسخ، علاوه بر معنی‌داری اثر بلوک (001/0 = P) و سن (001/0 = P)، معنی‌داری در شرایط یادگیری (003/0 = P) نیز مشاهده شد. همچنین، عملکرد آزمودنی‌های گروه‌های سنی مختلف در دو بلوک ابتدایی روز دوم نسبت به انتهای روز اول، در دو مؤلفه زمان (001/0 = P) و دقت پاسخ (001/0 = P) بهتر بود که نشان دهنده تحکیم کلی یادگیری حرکتی می‌باشد. نتیجه‎گیری: نتایج نشان داد که بین گروه‌های سنی مختلف، در اکتساب و تحکیم زمان و دقت پاسخ تکلیف توالی حرکتی، تغییرات عملکردی وابسته به سن وجود دارد. از طرف دیگر، اجزای مختلف حرکت (دقت و سرعت) به شیوه‌های متفاوتی اجرا، تثبیت و تحکیم می‌شوند. این موضوع باید هنگام آموزش و انجام مداخلات آموزشی و توان‌بخشی مرتبط با کودکان و نوجوانان مورد توجه قرار گیرد.

Title: تدوین برنامه درمانی «اوتیسم یکپارچه با خانواده» و مقایسه اثربخشی آن با «پرنده کوچولو» بر کاهش استرس والدگری والدین کودکان مبتلا به اوتیسم

http://jrrs.mui.ac.ir/index.php/jrrs/article/view/2425

مقدمه: اوتیسم یکی از رایج‌ترین اختلالات عصبی- رشدی محسوب می‌شود که میزان استرس والدگری را به شدت افزایش می‌دهد. کاهش استرس والدگری، بر درمان کودک تأثیر مثبتی دارد. بر همین اساس، مطالعه حاضر با هدف تدوین برنامه آموزش والدین مؤثر بر کاهش استرس والدگری والدین کودکان مبتلا به اوتیسم انجام شد. مواد و روش‌ها: بخش اول این مطالعه از نوع کیفی و حاصل سه دسته اطلاعات شامل مصاحبه عمقی با 11 متخصص، مصاحبه نیمه ساختار یافته با 30 والد و مرور 101 مقاله بود. بخش دوم مطالعه از نوع آزمایشی، همراه با گروه شاهد و گروه بدون مداخله بود که در سه مرحله پیش‌آزمون، پس‌آزمون و پیگیری انجام گرفت. بدین منظور، 42 والد کودک مبتلا به اوتیسم از میان والدین این کودکان که در زمان انجام پژوهش از مرکز تهران اوتیسم خدمات توا‌ن‌بخشی دریافت می‌کردند، انتخاب شدند و به صورت تصادفی در سه گروه قرار گرفتند. ابزار مورد استفاده پژوهش، شاخص استرس والدگری Abidin (Parenting Stress Index یا PSI) بود. داده‌ها با استفاده از آزمون تحلیل کوواریانس و تحلیل واریانس یک‌طرفه در نرم‌افزار SPSS مورد تجزیه و تحلیل قرار گرفت. 05/0 > P به عنوان سطح معنی‌داری داده‌ها در نظر گرفته شد. یافته‌ها: بر اساس یافته‌های مطالعه حاضر، برنامه درمانی اوتیسم یکپارچه با خانواده، بر تقویت‌گری (032/0 = P)، خلق (010/0 = P)، پذیرندگی (013/0 = P)، سازش‌پذیری (004/0 = P) و نمره کل استرس کودک (004/0 = P) تأثیر معنی‌داری داشت. همچنین، با احساس صلاحیت (002/0 = P)، افسردگی (001/0 = P)، انزوای اجتماعی (002/0 = P)، دلبستگی (001/0 > P)، محدودیت نقش (001/0 = P)، سلامت والدین (003/0 = P)، نمره کل استرس والد (001/0 > P) و نمره کل استرس والد- کودک (001/0 > P) رابطه معنی‌داری را نشان داد. نتیجه‎گیری: در مقایسه با آموزش‌های والدین پیشین از جمله پرنده کوچولو که به طور عمده بر استرس قلمرو کودک مؤثر بودند، برنامه درمانی اوتیسم یکپارچه با خانواده به دلیل پرداختن به مسایل خاص هر خانواده و نیز جامع بودن، بر کاهش استرس قلمرو والد نیز مؤثر می‌باشد.

Title: تأثیر تمرینات پایه‌ ژیمناستیک بر ثبات‌ پاسچر کودکان دارای اختلال طیف اوتیسم

http://jrrs.mui.ac.ir/index.php/jrrs/article/view/2575

مقدمه: اختلالات تعادل، از جمله مشکلات حرکتی در کودکان مبتلا به اختلال طیف اوتیسم است. با توجه به این که حرکت ‌درمانی باعث بهبود توانایی‌های ‌حرکتی کودکان دارای اوتیسم می‌شود، این مطالعه با هدف بررسی تأثیر تمرینات پایه ‌ژیمناستیک بر ثبات ‌پاسچر کودکان دارای اوتیسم انجام شد. مواد و روش‌ها: مطالعه حاضر از نوع کارآزمایی بالینی همراه با پیش‌آزمون، پس‌آزمون و گروه شاهد بود و در آن 20 پسر دارای اختلال ‌طیف اوتیسم شرکت کردند. با استفاده از مقياس تشخيصی اوتيسم Gilliam (Gilliam Autism Rating Scale-II یا GARS) (ويرايش دوم)، افراد از لحاظ شدت اختلال در دو گروه 10 نفره مورد و شاهد همگن‌سازی شدند. برنامه تمرینی گروه تجربی شامل 12 هفته تمرینات پایه ‌ژیمناستیک به صورت سه‌ جلسه 45‌ دقیقه‌ای در هفته بود که در مرکز‌ اوتیسم‌ شهر اصفهان اجرا شد. به منظور ارزیابی ثبات ‌پاسچر، دستگاه صفحه نیرو مورد استفاده قرار گرفت. ‌داده‌ها با استفاده از آزمون تحلیل واریانس چند متغیره در سطح معنی‌داری 05/0 تجزیه و تحلیل گردید. یافته‌ها: در مرحله پس‌آزمون، طول نوسان کمتری در مرکز فشار کودکان گروه مورد (143 ± 657 میلی‌متر) نسبت به کودکان گروه شاهد (311 ± 1057 میلی‌متر) مشاهده شد (013/0 = P). تمرینات پایه‌ ژیمناستیک موجب کاهش معنی‌دار نوسان مرکز فشار در کودکان مبتلا به اختلال طیف اوتیسم شد (001/0 = P)، اما این تغییرات در گروه شاهد معنی‌دار نبود (050/0 < P). نتیجه‎گیری: به نظر می‌رسد که تمرینات پایه ژیمناستیک در کودکان دارای اوتیسم، باعث کاهش طول نوسان بدن در حالت ایستاده و بهبود ثبات پاسچر می‌شود

Title: تأثیر مداخله حسی و بار شناختی بر انتقال فاز نسبی در تکلیف هماهنگی دو دستی زنان سالمند

http://jrrs.mui.ac.ir/index.php/jrrs/article/view/2579

چکیده: هماهنگی دو دستی در انجام کارهای روزانه مانند پرتاب کردن، گرفتن و بسیاری از فعالیت‌های ورزشی بسیار مهم است. اجرای این اعمال به عوامل متفاوتی مانند ویژگی‌های حسی و توجهی وابسته می‌باشد. هدف از انجام تحقیق حاضر، بررسی اثر مداخله حسی و بار شناختی بر زمان انتقال فاز نسبی الگوی هماهنگی دو دستی برون مرحله در میان زنان سالمند بود. مواد و روش‌ها: در این مطالعه، 12 زن سالمند سالم و راست دست (843/2 ± 66 سال) از بین 24 داوطلب شرکت کننده در تحقیق، بر اساس معیارهای ورود انتخاب شدند. شرکت کنندگان حرکت خود را بر روی دستگاه هماهنگی دو دستی با فرکانس 5/0 هرتز و با الگوی هماهنگی برون مرحله شروع کردند. سپس، بعد از هر 5 ثانیه به تدریج 25/0 هرتز به فرکانس افزوده شد و تا جایی ادامه یافت که الگوی برون مرحله به الگوی درون مرحله تبدیل گردید. این تکلیف در دو حالت با بار شناختی و بدون آن و با دستکاری حواس بینایی، شنوایی و لامسه انجام گرفت. داده‌ها با استفاده از تحلیل واریانس 4 × 2 با اندازه‌گیری مکرر، تصحیح Bonferroni و آزمون Paired t مورد تجزیه و تحلیل قرار گرفت. یافته‌ها: بر اساس یافته‌ها، اثر اصلی بار شناختی (004/0 = P)، اثر اصلی دستکاری حواس (040/0 = P) و اثر تعاملی آن‌ها (02/0 = P) بر زمان انتقال فاز نسبی معنی‌دار بود. اثر دستکاری حواس در شرایط بدون بار شناختی نیز معنی‌دار به دست آمد (004/0 = P). در شرایط بدون بار شناختی، تنها مداخله در حس عمقی به صورت معنی‌داری (001/0 = P) بر زمان انتقال فاز نسبی تأثیر داشت. نتیجه‌گیری: نتایج نشان دهنده نقش بارز بار شناختی بر تکلیف مورد نظر بود. در شرایط بدون بار شناختی، حس عمقی اثر قوی‌تری را نسبت به بینایی و شنوایی داشت، اما در شرایط اعمال بار شناختی، اثر مداخله حواس از بین می‌رود. با توجه به تغییر زمان انتقال با تغییر قیود تکلیف و قیود محیطی، نتایج این پژوهش با دیدگاه سیستم‌های پویا هم‌راستا است.

Title: تأثیر سفتی کفی کفش در دو سرعت مختلف بر سفتی پا طی فاز اتکای دویدن مردان فعال

http://jrrs.mui.ac.ir/index.php/jrrs/article/view/2548

مقدمه: اثر کفی کفش با ویژگی‌های مختلف و در سرعت‌های مختلف دویدن، روی سفتی پایین تنه هنوز ناشناخته است. هدف از انجام مطالعه حاضر، بررسی اثر دو نوع کفی (نرم و نیمه سخت) در دو سرعت مختلف دویدن بر سفتی پا طی فاز اتکای دویدن مردان فعال بود. مواد و روش‌ها: در این مطالعه، 15 مرد بدون هیچ گونه سابقه آسیب در اندام تحتانی انتخاب شدند. از آزمودنی‌ها درخواست شد که با دو سرعت کنترل شده 2/0 ± 0/3 و 1/0 ± 0/5 متر بر ثانیه در شرایط کنترل و کفی (نرم و نیمه سخت)، بر روی صفحه نیرو که وسط یک باند 15 متری قرار داشت، بدوند. سینماتیک و سینتیک حرکت با استفاده از 5 دوربین ویدئو و یک صفحه نیرو اندازه‌گیری و محاسبه گردید. سفتی پا با تقسیم نیروی عکس‌العمل عمودی زمین بر تغییر طول پا به دست آمد. جهت تست فرضیات، آزمون ANOVA دوطرفه با اندازه‌گیری مکرر مورد استفاده قرار گرفت (050/0 ≥ P). یافته‌ها: اختلاف معنی‌داری بین دو نوع کفی در سفتی پا وجود داشت و کفی نیمه سخت، سفتی پا را به طور معنی‌داری افزایش داد (001/0 > P)، اما این اختلاف به سرعت دویدن بستگی نداشت (990/0 = P). همچنین، تفاوت معنی‌داری در سفتی پا در دو سرعت مختلف مشاهده نشد (632/0 = P). نتیجه‎گیری: به نظر می‌رسد که با افزایش سفتی کفی کفش، ممکن است سفتی پا نیز افزایش یابد. همچنین، اثر کفی بر سفتی پا به سرعت دویدن بستگی ندارد و سفتی، با دویدن در سرعت پایین تا متوسط ثابت می‌ماند.

Title: عملكرد افراد مبتلا به مالتيپل اسكلروزيس در تست‌هاي تعادلي به دنبال كينزيوتيپينگ تسهيلي و مهاري عضله گاستروكنميوس: مطالعه تصادفي يك سوكور مقدماتی

http://jrrs.mui.ac.ir/index.php/jrrs/article/view/2619

مقدمه: مالتیپل اسکلروزیس (Multiple sclerosis یا MS) بیماری از بین برنده میلین سیستم اعصاب مرکزی می‌باشد که در ایران به صورت قابل توجهی رو به افزایش است. مشکلات ایجاد شده در سیستم عصبی به همراه آسیب سیستم حسی، منجر به اختلالات تعادلی، زمین خوردن و شکستگی می‌شود. کینزیوتیپینگ یکی از روش‌های فیزیوتراپی به شمار می‌رود که اثر آن بر روی تعادل در گروه‌های مختلف بررسی شده است. هدف از انجام مطالعه حاضر، بررسی تأثیر فوری کینزیوتیپینگ مهاری و تسهیلی بر روی تعادل افراد مبتلا به MS بود. مواد و روش‌ها: در این تحقیق یک سوکور، داوطلبان دارای معیارهای شرکت در مطالعه به صورت تصادفی به دو گروه تقسیم شدند. یک گروه، کینزیوتیپینگ تسهیلی و گروه دیگر، کینزیوتیپینگ مهاری را برای عضلات گاستروکنمیوس دریافت کردند. قبل و 30 دقیقه بعد از چسباندن تیپ، آزمون‌های تعادلی برخواستن و راه رفتن (Timed Up and Go یا TUG) و دسترسی عملکردی (Functional reach) انجام گرفت. محقق ارزیابی کننده تعادل، نسبت به روش چسباندن تیپ ناآگاه بود. یافته‌ها: تجزیه و تحلیل درون گروهی با استفاده از آزمون Paired t قبل و بعد از درمان، تفاوت معنی‌داری را در نتایج آزمون‌های TUG (001/0 > P) و دسترسی عملکردی (001/0 > P) در گروه تیپ مهاری (001/0 > P) و تیپ تسهیلی (020/0 = P) نشان داد. در مقایسه بین دو گروه نیز از آزمون Independent t قبل و بعد از درمان استفاده گردید که تفاوت معنی‌داری بین دو گروه مشاهده نشد (050/0 < P). نتیجه‌گیری: به نظر می‌رسد که استفاده از کنزیوتیپینگ عضله گاستروکنمیوس به هر دو روش مهاری و تسهیلی، باعث بهبود فوری تعادل افراد مبتلا به MS می‌شود.

Title: ارتباط شاخص معلولیت صوت و سابقه خدمت در آسیب شناسان گفتار و زبان

http://jrrs.mui.ac.ir/index.php/jrrs/article/view/2662

مقدمه: آسیب شناسان گفتار و زبان به عنوان یکی از کاربران حرفه‌ای صوت، از صدای خود به عنوان ابزار اصلی کارشان استفاده می‌نمایند. چنانچه راه‌های طبیعی نگه داشتن صوت را اعمال نکنند، به عوارض استفاده بد و نادرست صدا مبتلا خواهند شد. مطالعه حاضر با توجه به اهمیت و نقش صوت در عملکرد شغلی آسیب شناسان گفتار و زبان صورت گرفت. هدف آن تعیین کیفیت زندگی مرتبط با معلولیت صوت دو گروه از آسیب شناسان گفتار و زبان با سنوات خدمت کمتر و بیشتر از ۱۰ سال بود. مواد و روش‌ها: ۳۸ آسیب‌شناس گفتار و زبان شهر اصفهان به روش نمونه‌گیری در دسترس انتخاب شدند و به صورت هدفمند در دو گروه با سنوات خدمت کمتر و بیشتر از ۱۰ سال قرار گرفتند. پرسش‌نامه VHI (Voice handicap index) با هدف بررسی کیفیت زندگی مرتبط با معلولیت صوت توسط آن‌ها تکمیل گردید. میانگین نمرات آزمون VHI دو گروه با استفاده ازآزمون Mann-whitney تجزیه و تحلیل شد. آزمون ضریب همبستگی Spearman جهت بررسی همبستگی نمره کلی VHI و سه زیرآزمون عملکردی، عاطفی و فیزیکی با سنوات خدمت مورد استفاده قرار گرفت. یافته‌ها: نمره کلی VHI دو گروه کمتر از نمره برش پرسش‌نامه بود. همچنین، میانگین و انحراف معیار نمره کلی VHI و سه زیرآزمون گروه آسیب شناسان گفتار و زبان با سابقه بیشتر از ۱۰ سال کمتر از گروه آسیب شناسان با سابقه کمتر از ۱۰ سال به دست آمد (۰۵/0 > P). ارتباط معکوسی بین نمره کلی و نمرات زیرآزمون‌های VHI با سنوات خدمت آسیب شناسان گفتار و زبان وجود داشت، اما این ارتباط تنها بین نمره کلی و زیرآزمون عملکردی از لحاظ آماری معنی‌دار بود (۰۵/0 > P). نتیجه‎گیری: آسیب شناسان گفتار و زبان در ابتدای زندگی حرفه‌ای به دلیل تجربه کمتر ممکن است مواردی از استفاده بد و نادرست صدا را نشان دهند؛ اما با کسب تجربه بیشتر و ثبات بیشتر در وضعیت شغلی، به تأثیرات استفاده بد و نادرست صدا توجه بیشتری می‌کنند.

Title: تأثیر کفی بر درد، عملکرد و بیومکانیک افراد مبتلا به سندرم درد پاتلوفمورال: مطالعه مروری

http://jrrs.mui.ac.ir/index.php/jrrs/article/view/2569

مقدمه: سندرم درد پاتلوفمورال (Patellofemoral pain syndrome یا PFPS) یا سندرم درد قدام زانو (Anterior knee pain syndrome)، یکی از شایع‌ترین اختلالات اسکلتی- عضلانی در میان افراد بالغ و جوان محسوب می‌شود. استفاده از انواع ارتزهای پا از جمله کفی، در درمان این سندرم بسیار رایج است. نتایج موجود در رابطه با تأثیر این نوع ارتزها بر درد و بیومکانیک مبتلایان به PFPS متفاوت می‌باشد. هدف از انجام مطالعه حاضر، بررسی تأثیر کفی طبی بر درد، عملکرد و بیومکانیک افراد بالغ مبتلا به PFPS بود.  مواد و روش‌ها: جستجوی گسترده‌ای در پایگاه‌های اطلاعاتی IranMedex، Magiran، PubMed، ScienceDirect، Medline و ISI Web of Knowledge از سال 2000 تا 2015 انجام شد و کیفیت مقالات از طریق ابزار Black and Downs مورد ارزیابی قرار گرفت. یافته‌ها: از میان 117 مقاله حاصل از جستجو در پایگاه‌های فارسی و انگلیسی بر اساس واژه‌های کلیدی و بررسی عنوان، سرانجام 12 مقاله با توجه به معیارهای ورود به پژوهش، جهت ارزیابی نهایی انتخاب شد. نتیجه‌گیری: نتایج مطالعه حاضر نشان داد که استفاده از اشکال مختلف کفی، می‌تواند منجر به کاهش درد و بهبود عملکرد در افراد مبتلا به PFPS شود. با این وجود، تأثیر چنین ارتزهایی در بهبود علایم پاتلوفمورال افرادی که دارای پرونیشن پا هستند، بیشتر است. تأثیر کفی بر کینتیک و کینماتیک مفصل پاتلوفمورال ناچیز گزارش شد.
